# Effective strategies for prevention, control, and treatment of obesity in primary health care setting for adolescents, adults, and elderly people: A protocol for systematic review and meta-analysis

**DOI:** 10.1097/MD.0000000000010925

**Published:** 2018-06-01

**Authors:** Emanuele Souza Marques, Tatiana Henriques Leite, Catarina Machado Azeredo, Diana Barbosa Cunha, Eliseu Verly Júnior

**Affiliations:** a Department of Epidemiology, Institute of Social Medicine, Rio de Janeiro State University, Rio de Janeiro; b School of Medicine, Federal University of Uberlandia, Minas Gerais, Brazil.

**Keywords:** clinical trial, obesity, primary health care, review

## Abstract

**Background:**

It is unquestionable that obesity is a global epidemic and one of the main public health problems in the world. The management of obesity in Primary Health Care has an important role if being considered the magnitude and serious consequence of this problem. Despite this, there is no effective standard protocol for the treatment of this disease. Studies that synthesize and assess the effectiveness of strategies for prevention, control, and treatment of obesity in Primary Health Care setting are still scarce. The objective of this study is review and synthesize study evidence for obesity management strategies among adolescents, adults and elderly developed at the Primary Health Care worldwide.

**Methods:**

Seven electronic databases (Medline, Lilacs, Embase, Psycinfo, Cochrane, WHOLIS and Open Gray) will be searched with no date limit for identification of clinical trials examining the effectiveness of prevention, control and treatment of obesity in Primary Health Care. As primary outcome will be changes in body weight. As secondary outcomes will be body mass index, body adiposity, waist circumference, and waist-hip ratio. Two independent authors will perform the selection of studies, data extraction, and the assessment of risk of bias.

**Results:**

The results will be published in a peer-reviewed journal.

**Conclusion:**

This systematic review will be first to synthesize scientific evidence for obesity management strategies at Primary Health Care among adolescents, adults, and elderly. The review will benefit healthcare professionals and policymakers.

**Ethics and disseminations:**

Ethical approval is not required in this study because the data used include peer-reviewed publications, which do not comprise any information that could identify subjects.

**Trial registration number:**

PROSPERO (CRD42018092416).

## 1 Introduction

It is unquestionable that obesity is one of the main public health problems in the world, being considered a global epidemic.^[1–4]^ According to the World Health Organization, the prevalence of obesity has almost tripled since 1975.^[3]^ In the last decades, the prevalence of overweight and obesity in childhood and adolescence has been increasing significantly in high, middle, and low-income countries.^[5–11]^ In 2016, over 381 million children and adolescents were overweight or obese. In the same year, over 650 million (13% of the world population in this age group) adults were obese.^[3]^

Due to its high magnitude and serious consequences, the management of obesity in health services is a key element to address this problem. Primary Health Care stands out as an important setting for the development of prevention, control, and treatment of obesity due to characteristics such as universal accessibility, coverage on the basis of need, longitudinally of care, and intersectoral approaches.^[12,13]^ The health professional must carry out individual and/or collective interventions that involve social, psychological, genetic, clinical and dietary issues involved in this issue, as well as encourage and support the adoption of healthy living habits (such as healthy eating and regular practice of physical activity).^[2,13,14]^

Studies that synthesize and assess the effectiveness of strategies developed in primary health care for obesity management are still scarce.^[15–17]^ The systematic review of Seburg et al^[17]^ aimed to identify randomized controlled trials of primary care-based obesity intervention in children and adolescents. This review found only 4 randomized controlled trials of primary care-based intervention specifically designed for adolescents. The systematic reviews of Barnes and Ivezaj^[15]^ and Mitchell et al^[16]^ analyzed only a specific type of intervention (dietetic consultations and motivational interview) in primary health care to support adults. These studies reinforce the need for more systematic reviews and meta-analyses to evaluate the effectiveness of prevention, control, and treatment of obesity developed in Primary Health Care.

Thus, the main objective of this study is to systematically review and synthesize study evidence for obesity management strategies among adolescents, adults, and elderly developed at the primary health care worldwide.

## 2 Methods

### 2.1 Inclusion and exclusion criteria

The selection of the studies included in the review will be done using the following inclusion criteria: articles available in English, Spanish, or Portuguese without limitation on the period of publication; obesity prevention, control, and/or treatment interventions; randomized controlled trial (pilot or full study); intervention delivered in primary health care setting; target audience for the intervention: adolescents, adults, or elderlies.

#### 2.1.1 Type of studies

All randomized clinical trials that analyze the impact of obesity prevention, control, and/or treatment interventions in primary health care published in peer reviews journal.

#### 2.1.2 Types of participants

Those studies evaluating adolescents, adults, and elderly population will be included in investigation.

#### 2.1.3 Types of comparator

Will be considered as comparator: no treatment group, placebo, or alternative therapy.

#### 2.1.4 Types of intervention

Any intervention study aiming to assess the effectiveness of intervention to prevent, control, or treat obesity developed in Primary health care.

#### 2.1.5 Type of outcome measure

The primary outcome will be body weight and body mass index (BMI). Secondary outcomes will be body adiposity, waist circumference, and waist–hip ratio.

### 2.2 Information sources and search strategy

#### 2.2.1 Electronic searches

Seven databases will be accessed (Medline, Lilacs, Embase, Psycinfo, Cochrane, WHOLIS, and Open Gray); the last 2 were specific to search “gray literature” The search will be conducted on Medline and adapted for the other databases, according to the following search strategy: (((((“obesity”[MeSH Terms] OR “obesity”[All Fields]) OR (“overweight”[MeSH Terms] OR “overweight”[All Fields]) OR (“body mass index”[MeSH Terms] OR (“body”[All Fields] AND “mass”[All Fields] AND “index”[All Fields]) OR “body mass index”[All Fields]))) AND ((((clinical[Title/Abstract] AND trial[Title/Abstract]) OR clinical trials as topic[MeSH Terms] OR clinical trial[Publication Type] OR random∗[Title/Abstract] OR random allocation[MeSH Terms] OR therapeutic use[MeSH Subheading])))) AND ((“primary health care”[MeSH Terms] OR (“primary”[All Fields] AND “health”[All Fields] AND “care”[All Fields]) OR “primary health care”[All Fields] OR (“primary”[All Fields] AND “care”[All Fields]) OR “primary care”[All Fields]))) AND ((“adult”[MeSH Terms] OR “adult”[All Fields] OR “eldery”[All Fields] OR “eldery”[MeSH Terms] OR “adolescent”[MeSH Terms] OR “adolescent”[All Fields] OR “teenage”[MeSH Terms] OR “teenage”[All Fields])).

#### 2.2.2 Searching other resources

Manual searches will also be conducted in the reference sections of all selected studies, as well as in Google Scholar.

### 2.3 Selection of studies

All identified references will be exported from databases and archived in the EndNote X6 program.^[18]^ Two independent researchers will perform the selection of scientific articles, in 2 stages. In the first stage, articles will be selected based on titles and abstracts, according inclusion criteria aforementioned. The next stage will involve reading the full text to confirm the eligibility criteria. Disagreements between reviewers will be resolved by consensus. The whole process of study selection is summarized in the PRISMA flow diagram.^[19]^

### 2.4 Data extraction and management

The extraction of information will be carried out by 2 researchers independently. Other 2 reviewers will perform a crosscheck of the first 15 references extracted to assure accuracy and completeness of data. For studies with multiple publications, all publications identified in the review will be used for data extraction. The data extracted will be done in a structured form. The extracted data will include: general information (author, title, publication year, journal, randomization, blinding); participants (sample size, general characteristics, comorbidities); intervention (type of intervention and duration); control (no treatment, placebo, or alternative therapy); outcomes (reported outcomes). Intervention effectiveness will be evaluated according to Seburg et al.^[17]^ Interventions will be considered effective if there is a significant difference in weight and BMI outcomes. Thus, for the analysis of effectiveness only the studies that performed the intention-to-treat analysis will be considered.

Information extracted per article and researchers will be compared and any differences will be resolved by referencing back to the original publication. The authors of the respective studies will be asked for any missing information required.

### 2.5 Assessment of risk of bias in included studies

Quality assessment will be performed for each outcome of each study by 2 reviewers independently. Disagreement will be resolved first by discussion and then by consulting a third reviewer to make a final decision.

Assessment of study quality will follow the recommendations of “The Joanna Briggs Institute Reviewers Manual 2015”—MAStARI Critical Appraisal Checklist, using specific criteria for intervention studies. In this questionnaire there are 10 questions about the method of original study. For all question it is possible mark “Yes,” “No,” or “Unclear”^[20]^:

1) Is the assignment to treatment groups truly random?
2) Are participants blinded to treatment allocation?
3) Is allocation to treatment groups concealed from the allocator?
4) Is the outcome of people who withdrew described and included in the analysis?
5) Are those assessing the outcome blind to treatment allocation?
6) Are the control and treatment groups comparable at entry?
7) Are groups treated identically other than for the named intervention?
8) Are outcomes measured in the same way for all groups?
9) Are outcomes measured in a reliable way?
10) Is appropriate statistical analysis used?

After the analyses considering all publication, sensitivity analyses will be performed, and studies with important methodological limitations will be excluded.

In addition, the publication bias or other asymmetry in published results due to quality problems will be assessed by the funnel plot and the Egger tests.^[21]^

### 2.6 Data synthesis

At first, will be presented a descriptive synthesis of the main findings of the included studies. A summary table will depict variables, such as the type of intervention, the characteristics of the target population, the type of outcome, and the content of the intervention. If possible, will be combined the data through meta-analysis using fixed and/or random-effect analysis as statistical convenience.^[21]^ The analysis will be done considering the type of intervention (prevention, control, or treatment) and population age group (adolescent, adult, and elderly). Data will be analyzed using the *metan* commands in Stata 14.0 (Stata Statistical Software: Release 14. College Station, TX: StataCorp LP).^[22]^

#### 2.6.1 Assessment of heterogeneity

Heterogeneity between studies will initially be evaluated by visual inspection of forest-plots. Statistical significance of heterogeneity will be assessed by *Q*-test while the proportion of true heterogeneity to total variance calculated by the Higgins *I*^2^ statistic. This statistic will be considered heterogeneity *I*^2^ ≥ 50%.^[21]^

If significant heterogeneity is present, subgroup analyses will be included. Variable as sex, comorbidities, economic and social situation will be investigated. Models of meta-regression will be conducted, including variables related aiming at identifying possible sources of inconsistency—heterogeneity—between the studies.

### 2.7 Ethical and dissemination

Ethical approval is not required in this study because the data used include peer-reviewed publications, which do not comprise any information that could identify subjects in the original studies.

### 2.8 Registration

The protocol of this systematic review has been registered on PROSPERO (CRD42018092416). This systematic review protocol was reported using the Preferred Reporting Items for Systematic Reviews and Meta-Analyses (PRISMA-P) statement guidelines.^[23]^

## 3 Discussion

This systematic review may provide a detailed summary of current evidence regarding the efficacy of prevention, control and treatment of obesity in adolescents, adults, and the elderly in Primary Health Care setting. The results of this review will be widely disseminated through peer-reviewed publications and conference presentations. Thus, the result will be informative in terms of patient care, may guide decision-making in relation to the development of more effective prevention, control, and treatment actions in this population assisted in Primary Health Care.

## Author contributions

**Methodology:** Emanuele Marques, Tatiana Leite, Catarina Azeredo, Diana Cunha, Eliseu Verly Júnior.

**Project administration:** Emanuele Marques.

**Writing – original draft:** Emanuele Marques, Tatiana Leite, Catarina Azeredo, Diana Cunha, Eliseu Verly Júnior.

**Writing – review & editing:** Emanuele Marques, Tatiana Leite, Catarina Azeredo, Diana Cunha, Eliseu Verly Júnior.
